# Large scale analysis of amino acid substitutions in bacterial proteomics

**DOI:** 10.1186/s12859-016-1301-5

**Published:** 2016-11-08

**Authors:** Dmitry Ischenko, Dmitry Alexeev, Egor Shitikov, Alexandra Kanygina, Maja Malakhova, Elena Kostryukova, Andrey Larin, Sergey Kovalchuk, Olga Pobeguts, Ivan Butenko, Nikolay Anikanov, Ilya Altukhov, Elena Ilina, Vadim Govorun

**Affiliations:** 1 Research Institute of Physical Chemical Medicine, Malaya Pirogovskaya, 1a, Moscow, 119435 Russian Federation; 2 Moscow Institute of Physics and Technology, Institutskiy pereulok, 9, Dolgoprudny, 141700 Russian Federation

**Keywords:** Spectral library, SAP

## Abstract

**Background:**

Proteomics of bacterial pathogens is a developing field exploring microbial physiology, gene expression and the complex interactions between bacteria and their hosts. One of the complications in proteomic approach is micro- and macro-heterogeneity of bacterial species, which makes it impossible to build a comprehensive database of bacterial genomes for identification, while most of the existing algorithms rely largely on genomic data.

**Results:**

Here we present a large scale study of identification of single amino acid polymorphisms between bacterial strains. An ad hoc method was developed based on MS/MS spectra comparison without the support of a genomic database. Whole-genome sequencing was used to validate the accuracy of polymorphism detection. Several approaches presented earlier to the proteomics community as useful for polymorphism detection were tested on isolates of *Helicobacter pylori*, *Neisseria gonorrhoeae* and *Escherichia coli*.

**Conclusion:**

The developed method represents a perspective approach in the field of bacterial proteomics allowing to identify hundreds of peptides with novel SAPs from a single proteome.

**Electronic supplementary material:**

The online version of this article (doi:10.1186/s12859-016-1301-5) contains supplementary material, which is available to authorized users.

## Background

To date, the common method of proteins and peptides identification in tandem mass spectrometry (MS/MS)-based proteomics, employs a genome sequence-dependent approach: query spectra are searched against a database constructed from the theoretical spectra derived from peptides identified in silico in the annotated genome of the corresponding species.

However, this method has shown insufficient accuracy when applied to the species including representatives with highly variable genomes and is unable to detect translation errors accumulated due to the malfunctioning of protein synthesis machinery of a cell. The efficiency is also negatively affected if the genome sequence of the examined species is not available and the database is constructed from the peptides of related species belonging to the same taxonomic group of higher order (genus, family or class). Any polymorphisms between the genomes including single nucleotide polymorphism (SNPs) and corresponding single amino acid polymorphism (SAPs) result in substantially different theoretical spectra, which further complicates the problem of peptide identification more complicated [1]. Experimental spectra are affected in a similar way by post-translational modifications (PTM)[2].

To overcome these problems, some algorithms use a set of all possible modified peptides. One of the solution for SAPs identification is an approach demonstrated in MSMSpdbb [3], a protein database which comprises all known peptide variants. The database is constructed from all known homologous peptide sequences for a given species merged into synthetic sequences. In the similar way act approaches that use data from public repositories of genetic variants or sample-specific NGS data and generate variant-containing databases and thereby enable detection of SAP-containing peptides [4]. These approaches are more flexible but still does not allow to identify peptides not present in the database.

There are several approaches allow to identify the spectra corresponding to the peptides that differ from those in the theoretical database, considered all the possible sequences of modified peptides as a database for searching [5–7]. As this approach allows exhaustive test of all amino acid substitutions and greatly expands search space, statistical significance for the variant identifications is not easily evaluated [8].

Another class of methods is based on the comparison of query spectra with the annotated reference spectra, so-called spectral libraries [9]. This approach was shown to perform better than the genome sequence-dependent algorithms in terms of sensitivity and specificity due to the employment of data on real MS2 peaks and their spectral intensities and decrease in the total number of sought variants [10]. Similarity measures are usually calculated as a function based on the spectral angle between the peaks. However, this approach still does not allow to detect the peptides not present in the spectral library.

As in the case of genome sequence-dependent approaches, there are several solutions which allow to identify the spectra corresponding to the peptides with PTM based on the modification of the spectra from spectral libraries. Notably, these methods are able to identify both specific and potential PTMs (so-called blind PTM search). Among these are pMatch [11] and Tier-Wise scoring [12] methods. These algorithms have not been tested on a large number of peptides with SAPs yet.

In this paper we describe SAP identification method based on the comparison investigated spectra with spectral library. The algorithm is designed specially for SAP identification and consists of several steps, including PTM filtering.

The research was conducted on MS/MS peptide spectra of highly variable bacterial species including *H. pylori*, *N. gonorrhoeae* and *E. coli*. The training of the algorithm is performed on a several hundreds of thousands of *H. pylori* spectra and on 1,000 different peptide variants. The results were compared to both genome sequence-dependent and library-based approaches and validated using *H. pylori* and *N. gonorrhoeae* spectra. To validate results bacterial isolates were sequenced and genomes were assembled and annotated, algorithm identifications were compared to identifications against protein database obtained from annotation of corresponding genomes. Additionally, using open MS/MS datasets different host-specific *E. coli* strains were analyzed in order to divide them into clusters according to the host of origin using the SAP data and phylogenetic methods.

## Methods

### Algorithm description

Algorithm for spectra comparison is based on the measure of spectral angle between two spectra [13, 14]. Briefly, each spectrum is represented as a multidimensional vector. The probability of the fact that two spectra correspond to the same peptide depends on a cosine similarity between these vectors (Additional file 1: Section 2.1). In the case of detection of spectra corresponding to the peptides with a SAP between them, we reconstruct the estimated spectrum alteration due to the SAP and finally calculate the angle between the modified spectra from the annotated spectral library and candidate spectra. SAP identification requires two sets of spectra:

1. Spectral library (SL) - the set of spectra acquired from a reference organism with known genome. Each spectrum in the SL is annotated using the peptide identification program, in our case Mascot.
2. SAP candidates (SP) - the set of spectra (usually from the same species as used for SL) where we would expect to find the spectra of peptides with SAP relative to the peptides of reference organism.

**Fig. 1:**
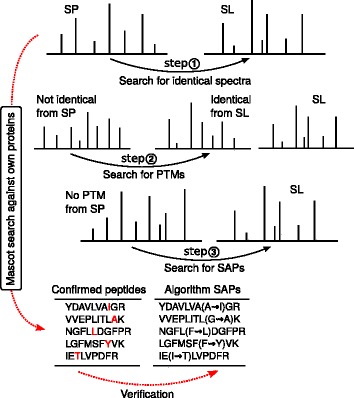
Major steps of the *speptide* algorithm

There are several major steps in the algorithm (Fig. 1).

① **Filtering identical spectra**. Removing the spectra similar to those in SL from the candidate library SP. To identify the spectra of identical peptides, we compare the spectra with equal (within a predefined accuracy up to MS1) parent mass. For two spectral sets (SP and SL) with a given *Δ*_*MS*1_ the candidate pairs of spectra are defined as those with |*M* *S*1_*SP*_−*M* *S*1_*SL*_|≤*Δ*_*MS*1_. Each spectrum from SP is assigned to a spectrum from SL corresponding to the minimal angle between two vectors. We can further use the cut-off value for spectral angle to sort out the identical spectra.
② **PTM Filtration**. The influence of SAP on the spectra is similar to that of PTM. We use an additional step of true PTM filtration to filter out probable PTMs of the peptides occurring in SP. Those pairs could be found using the spectra identical between SP and SL (determined at the first step) as a matrix for mass shift search. It allows to scan SP spectra for the mass shifts equal to PTMs. The same approach was used as an approach for SAP identification described below. The spectra identified as potential PTMs are removed from the SP set.
③ **Amino acid substitution search**. The list of possible single amino acid modifications is constructed under the assumption that amino acid has changed due to a single nucleotide mutation. For two spectral sets (SP and SL) with a given *Δ*_*MS*1_, the candidate pairs of spectra are defined as those with ∃*D*_*δ*_∈{*D*}:*D*_*δ*_−*Δ*_*MS*1_≤*M* *S*1_*SL*_−*M* *S*1_*SP*_≤*D*_*δ*_+*M* *S*1, where {*D*} is a set of mass differences for selected amino acid substitutions. A spectrum from SL is transformed according to the chosen method. Each spectrum from SP is assigned to a spectrum from SL corresponding to the minimal angle between the two vectors.

### Bacterial strains

Two laboratory strains (J99 and 26695) and three clinical isolates (A45, H13-1 and E48) of *Helicobacter pylori*, as well as one laboratory strain (FA1090) and two clinical isolates (i19.05 and n01.08) of *Neisseria gonorrhoeae* were included in the analysis. Clinical isolates of *N. gonorrhoeae* and *H. pylori* were collected as a part of the previous studies [16, 17]. Laboratory strains of *N. gonorrhoeae* and *H. pylori* were obtained from the ATCC collection [18]. Additionally, MS/MS spectra for 73 *Escherichia coli* strains were obtained from previous study [15].

### Culture growth conditions

*H. pylori* strains were cultured on Columbia agar (BioMerieux, France) containing 10 % inactivated horse serum (PAA Lab., Austria), 5 % yeast dialysate (Gamaleya Research Institute of Epidemiology and Microbiology RAMS) and selective antibiotic mix ”Dent” (Oxoid, England) at 37 °C and 5 % *C* *O*_2_. *N. gonorrhoeae* strains were cultivated on GC agar (Becton Dickinson, USA) supplemented with 1 % IsoVitaleX (Becton Dickinson, USA), at 37 °C and 5 % *C* *O*_2_. All bacterial cells were collected into Hanks buffer and then collected by centrifugation at 6,000 RPM for 10 min at 4 °C.

### Genomic analysis

Genomes of *H. pylori* clinical isolates were sequenced using GS Junior genome sequencer (Roche 454 Life Science, USA) for the strain A45 and Ion PGM sequencer system (Applied Biosystems, USA) for the strains E48 and H13-1. Whole genome sequencing of the *N. gonorrhoeae* clinical isolates was performed on GS FLX+ genome sequencer (Roche 454 Life Science, USA) using a standard protocol for a ”shotgun” genomic library. The raw reads were subject to correction using SAET 3, and Newbler v.2.6 (Roche 454 Life Science, USA) was used for assembly *de novo*. To yield a consistent annotation across all genomes in the study, the genomes were reannotated using a Prokka-based workflow [19]. To include possibly missing annotations of the WGS genomes, Artemis [20] (release 16) was used to annotate all the ORFs including those located at the ends of the contigs

### Proteomic analysis

The bacterial cells were suspended as described in [21]. The LC-MS was performed on a TripleTOF 5600+ (Sciex, USA) mass-spectrometer operating in in a data-dependent mode with a NanoSpray III ion source (Sciex, USA) coupled to a NanoLC Ultra 2D+ nano-HPLC system (Eksigent, USA) configured as described in [22]. Raw data files in WIFF and.D file formats were converted to the Mascot generic format (MGF file format) using AB SCIEX MS Data Converter version 1.3 and Compass Data Analysis 4.2 (Build 383.1), respectively. Mascot 2.2.07 was used for the identification with the following parameters: MS1 tol: 10 ppm, MS2 tol: 0.5 Da, variable modifications: Oxidation(M) and Carbamidomethylation(C), trypsin specificity with 1 missed cleavages allowed. The Paris proteomics guidelines for identification were used [23]. Decoy searches were implemented by database construction with reversed proteins.

### Algorithm implementation

Algorithm was implemented as an ad hoc software program *speptide* written in C/C++ available with full help file and examples at: https://github.com/dimaischenko/speptide.

### Data analysis

Analyses were conducted in R [24] with the “phangorn” [25], and “data.table” [26] packages. Spectral library creation and filtration were performed with an ad hoc perl scripts included in *speptide* source code.

## Results and discussion

### Datasets explanation

**Fig. 2:**
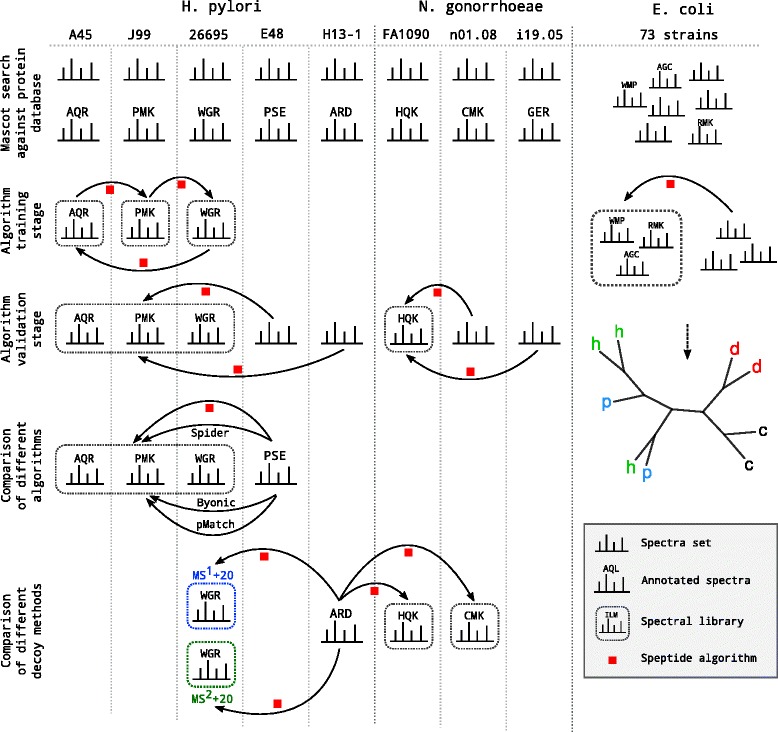
Schematic representation of the major steps of the study and the data used

We used mass spectrometry data for three bacterial species: *H. pylori*, *N. gonorrhoeae* and *E. coli*. The description of the datasets used at the different steps of the analysis is provided below. The schematic representation of these stages is shown in Fig. 2.

1. *H. pylori*. Three strains (A45, J99 and 26695) were used for algorithm training and parameter estimation; the remaining two strains (E48 and H13-1) were used for SAP search against the combined spectral library (A45, J99 and 26695) at the validation step.
2. *N. gonorrhoeae*. All three strains were used at the validation step. SAPs in strains n01.08 and i19.05 against strain FA1090 were identified.
3. *E. coli* (dataset of 73 strains isolated from 4 different hosts). The data was used to demonstrate the possibility to separate the host-specific strains into clusters according to their hosts basing on the identified SAPs.

Prior to the construction of the spectral libraries, algorithm training and validation, we performed the identification of the spectra for each bacterial species against a database constructed from the genes derived from the genome annotations of these species using Mascot. This allowed us to estimate false and true positive identifications, specificity and sensitivity of the algorithm at each stage. We also estimated the highest possible number of SAPs identified for each search basing on the comparison of the peptide sequences of the identified spectra (see Tables 1 and 2 and Additional file 1: Section 1.3).

**Table 1:** Results of Mascot identification of MS spectra against the annotated genomes of the species

Species	Strain	Genome NCBI	Total # of	# of identified	# of identified	Mascot threshold	Decoy threshold
		accession	spectra	spectra	unique peptides	score	score
*H. pylori*	A45	AMYU00000000	74800	15108	6606	8	8
*H. pylori*	26695	NC_018939	71801	14732	6737	8	8
*H. pylori*	E48	AYHQ00000000	58704	14655	6506	8	8
*H. pylori*	J99	NC_000921	68739	15279	6374	8	8
*H. pylori*	H13-1	AYUH00000000	64585	17554	7341	8	8
*N. gonorrhoeae*	i19.05	JFBA00000000	72851	24964	6374	9	6
*N. gonorrhoeae*	n01.08	JIBZ00000000	83672	32005	6761	9	6
*N. gonorrhoeae*	FA1090	NC_002946.2	44803	11891	5072	9	10

**Table 2:** Estimation of the number of possible SAP identifications based on the comparison of the whole set of peptides identified for each sample

Subject	# of spectra	# of peptides	Query database	# of spectra	# of peptides	# of peptides with	# of possible peptides
database	in subject	in subject		in query	in query	1 SAP detected	identifications with 1
	database	database		database	database	by genomes	SAP by proteomes
*H. pylori* 22695	13306	6012	*H. pylori* J99	13789	5747	4293	630
*H. pylori* A45	13657	5932	*H. pylori* 22695	13306	6012	3857	603
*H. pylori* J99	13789	5747	*H. pylori* A45	13657	5932	4308	502
*H. pylori*	40752	10759	*H. pylori* E48	12440	5529	3446	467
(A45, J99, 26695)							
*H. pylori*	40752	10759	*H. pylori* H13-1	15194	6402	3322	523
(A45, J99, 26695)							
*N. gonorrhoeae*	10854	4587	*N. gonorrhoeae*	29462	6606	764	66
FA1090			*i19.05*				
*N. gonorrhoeae*	10854	4587	*N. gonorrhoeae*	32815	6453	765	87
FA1090			*n01.08*				

### Algorithm training stage

At the training stage we used spectral sets from three strains of *H. pylori* - A45, J99 and 26695 - as a training dataset for the algorithm. Three pairwise comparisons were performed; in each of them, one sample is considered as a reference sample and the other - as a query sample: A45 → J99, J99 → 26695, 26695 → A45 (the second sample in each pair serving as reference). The training was performed for both the algorithm of identification of similar peptides and the SAP identification algorithm.

The initial spectra is not always appropriate for calculation of the spectral angle [27]. There are several approaches to the transformation of the initial spectra. It is known that the transformation of intensity values improves the results. It was also shown that limiting the number of peaks in comparison can improve the accuracy of the comparison. Different types of the considered peaks (*b*−, *y*−, *b*−*H*_2_ *O*, *b*−*N* *H*_3_, *y*−*H*_2_ *O*, *b*−*N* *H*_3_) and different methods of peak shifting (i.e. shift only the annotated, shift all peaks, duplicate peaks) in the reference spectrum and intensity transformation (ln*I*, $\sqrt {I}$, *I*) were applied; the number of the peaks from the reference and query spectra selected for further analysis was also varied. It should be noted that for each set of spectra we performed the search against the genes acquired from the annotation of the corresponding genomes with Mascot, and the results of the algorithms at the training stage were verified by comparison with these Mascot identifications. For each parameter values areas under ROC curves are calculated, and the number of true positive identifications is estimated for two FDR cut-offs 1 % and 5 %. The detailed description of all varied parameters and peaks shifting methods at the training stage are described in supplementary (Additional file 1: Sections 2.2 and 2.3). At the final stage of the algorithm training, the optimal parameter and cut-off values were selected for the algorithms of similar peptides search, PTM filtering and SAP identification.

### Algorithm validation stage

For the validation of the method and testing of the adjusted parameter values, we identified the peptides with SAPs in MS/MS spectra not used at the training stage and validated these identifications by standard MS/MS spectra identification from the corresponding genomes with Mascot.

Data on the *H. pylori* and *N. gonorrhoeae* species were used for this analysis. For each isolate, from 40 to 80 thousands spectra were acquired, and from 5 to 8 thousand peptides in each sample were identified.

For *H. pylori*, we used strains J99, 26695 and A45 as a reference set (SL). Strains H13-1 and E48 were used as the candidates for the SAP search (SP). For the peptide identification using Mascot, we have sequenced isolates A45, H13-1 and E48. The genomes were assembled and annotated. For each strain, the spectra set was identified using the database constructed from the corresponding genomes.

For *N. gonorrhoeae*, we used strain FA1090 as a reference (SL) and strains n01.08 and i19.05 - as candidates for the SAP search (SP). Strains n01.08 and i19.05 were sequenced, their genomes were assembled and annotated.

**Fig. 3:**
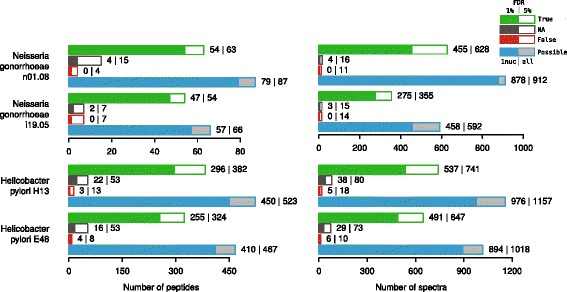
The number of the true and false identifications with *speptide* algorithm for the samples in the validation study with 5 % and 1 % FDR. Each diagram shows: (i) the number of the peptides (spectra on the right) identified for the selected isolate for two settings of FDR parameters, (ii) the number of NA identifications (see in the text), (iii) the number of false identifications, (iv) the total number of peptides (spectra on the right) with SAPs which could be detected

Results of the validation are shown in Fig. 3.

For all of the bacterial species, we managed to identify over the half of the peptides containing SAPs using parameters for FDR of 5 %. For *N. gonorrhoeae*, the total number of the identified SAPs was relatively small. There could be two explanations for this result: firstly, the total number of proteins in a genome is high relative to the total number of identified proteins and the fractions of the highly expressed proteins intersect slightly between the strains in SL and SP, and, secondly, there is a relatively small number of the polymorphisms between the genomes (Table 2). *H. pylori* has a higher total amount of peptides identified containing one SAP difference and serves as a good model for the case when the spectral library is large enough. The possibility to detect 60–80 % of the total number of the peptides with SAPs was observed. The detailed data for specificity and sensitivity is presented in Table 3.

**Table 3:** Sensitivity and specificity of SAPs identification for *H. pylori* and *N. gonorrhoeae* at the validation stage for different a priori FDR cut-offs

Strain	Sensitivity		Specificity	
	5 % FDR	1 % FDR	5 % FDR	1 % FDR
*H. pylori* E48	0.693	0.546	0.994	0.996
*H. pylori* H13-1	0.730	0.570	0.993	0.996
*N. gonorrhoeae* i19.05	0.818	0.712	0.996	0.997
*N. gonorrhoeae* n01.08	0.724	0.620	0.996	0.998

The SAP identification could result in a higher number of the proteins identified in the sample. According to general proteomics guidelines, two peptides have to be identified to claim a reliable identification of a protein. To estimate the protein number gain due to SAP detection, the following calculation was performed. For the spectra of *H. pylori* H13-1 and E48, a conventional identification was performed using a database constructed from three known genomes of 26695, J99 and A45 strains. The numbers of the identified peptides and proteins weres calculated. This modeled the situation of classical proteomics where one performs a search for an isolate with unknown genome using reference genomes. As protein identification requires 2 peptides per protein for reliability, the implementation of the *speptide* algorithm allows to add 10 proteins to the list of E48 proteins (692 proteins before SAP search) and 15 proteins to the list of H13-1 proteins (760 identified before SAP search); the detailed information is available in Additional file 2.

### Comparison of algorithms and approaches

To compare the efficiency and performance of SAP detection approaches, we selected several additional algorithms: Byonic [6], pMatch [11] and SPIDER [7] (PeaksStudio). Byonic and SPIDER are based on the search against a genomic database; SPIDER includes a feature of de novo assembly, and pMatch allows to identify PTM against a spectral library. We used the A45, J99 and 26695 strains as a reference set and isolate E48 - as a test set in comparison. We used a spectral database constructed from the MS/MS spectra of strains A45, J99 and 26695 for *speptide* and pMatch and a database of the sequences of all identified peptides of these strains for Byonic and SPIDER. Two of the algorithms (Byonic and pMatch) are intended for PTM search, so the results were post-processed. During the post-processing, only the mass shifts in parent mass, that could be explained by amino acid changes were selected. The results for all algorithms were filtered using 5 % FDR threshold. We used the Mascot search against genome of *H. pylori* E48 as a validation step for the results of selected algorithms. If a peptide sequence identified by the selected algorithm matched Mascot identification using the genome of *H. pylori* E48, the identification was considered true. In the cases when Mascot identified a different peptide, the match was considered false.

**Fig. 4:**
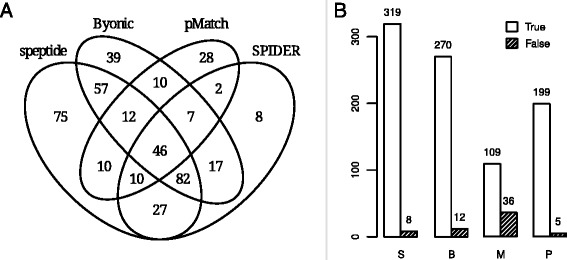
Comparison of the SAP identification in the peptides of *H. pylori* E48 against the combined *H. pylori* database (A45, J99, 26695) for four different algorithms: S - *speptide*, B - Byonic, P - SPIDER, M - pMatch. **a** Venn diagram for the unique peptides with SAP confirmed by Mascot search. **b** Numbers of true and false identifications for each algorithm

The detailed information on the algorithm parameters and computation efficiency is presented in the supplementary materials (Additional file 1: Section 3.3). Figure 4 shows the comparative analysis for the selected algorithms. Each of the algorithms allows identification of hundreds of peptides.

Noteworthy, *speptide* managed to identify over 75 peptides (Fig. 4 a) not identified by any other algorithm due to the usage of experimental spectrum for similar peptide as a reference instead of reconstructing a reference spectrum from sequence in the case of Byonic and SPIDER (even taking into account that *speptide* identifies only single nucleotide SAPs). In the case of pMatch, it is still designed for blind PTM search and was not fine-tuned to SAP identification. We suggest that, as there is a lack of studies analyzing a large number of SAPs, the existing algorithms are not readily optimized for the identification of such events.

The approach for mass shifts search described above is used in similar algorithms for PTM identification [28]. The major difference between PTM and SAP is the specificity of PTM. Most PTMs occur for a limited number of amino acids, i.e. phosphorylation only at Ser, Thr and Tyr or Met oxidation. SAP can basically occur at any position and any amino acid could be changed to any other. Therefore, the search space for SAP is wider than for the canonical PTM, but, at the same time, less than for a blind PTM search, and application of spectral algorithms capable of SAP identification requires a thorough testing and FDR estimation. We have shown that, for the case of SAP search in a single bacteria species proteome, the results are promising. While working with a high specificity, the algorithm is able to find up to 80 % of the SAPs.

### Unidentified peptides

In most of the performed searches, the *speptide* algorithm detects spectra that were not identified by Mascot, but, according to the algorithm, are candidates for SAP. As there is no evidence supporting these peptides, we designated these peptides as NA. Calculation of specificity and sensitivity of the algorithm was done as if NA peptides were false discoveries. However, we suspect the FDR calculated in this work is overestimated. Additional investigation on the nature of NA peptides was performed. There are hypotheses why peptides are not identified by Mascot, but identified by *speptide* algorithm:

1. Predicted SAPs in the peptides are false discoveries of *speptide* algorithm, the origin of spectra is unknown and the real peptide sequence is not contained in Mascot database.
2. The reference genome is misannotated, therefore, the true peptide sequence is not in the Mascot database, but peptides are contained in the sample.
3. Peptides in the sample contain PTM and SAP at the same time, and the total mass shift is equal to the mass shift from another SAP.

Described cases could lead the algorithm to the identification of a wrong SAP, while Mascot is unable to find peptide with PTM as peptide has SAP and, therefore, is absent from Mascot search space.

As a demonstration, we took the peptides against which the SAPs had been identified and aligned them using *tblastn* against the corresponding genomes of the analyzed strains (against which a SAP is supposed to be present). For most of the NA peptides, we can find a peptide in a genome which differs in 1 amino acid. Therefore, the *speptide* finds the peptide correctly, but misinterprets the amino acid change. In our case, the mass shift of an amino acid polymorphism proposed by *speptide* equals to the mass shift from the true amino acid (in genome) plus PTM. Overall, we find it impossible to distinguish the situation of 1 amino acid change and 1 amino acid change plus PTM using sparse MS/MS spectrum. The confirmation was performed using Mascot, where we searched the spectra of NA peptides against the true genome with additional variable modifications: Deamidation and Dimethylation. This resulted in the correct identification of 22 from 53 NA peptides in *H. pylori* H13-1, and 18 from 53 NA peptides in *H. pylori* E48 (Additional file 1: Section 3.1).

### Decoy database

Decoy database strategy was formulated by J.Elias [29]: “necessarily incorrect decoy sequences added to the search space will correspond with incorrect search results that might otherwise be deemed to be correct”. While for a convenient genomic database identification the approach of decoy database creation is well tested and usually implies shuffling amino acids in protein sequence [30], there is no optimal strategy for decoy spectral library creation. Several methods were suggested for the identification of spectra against spectral libraries on the basis of mixing peaks in decoy library spectra [31]. These approaches are also applicable for PTM search against spectral libraries [11, 12]. We employed these methods to estimate FDR of the SAP search by using an MS/MS spectral library of a different bacterial species as an additional decoy library. Indeed, it is created from the spectra from the same mass-spectrometry instrument, is filtered using a genomic database to filter out only the peptide matching the spectra, and, if the organisms are phylogenetically distinct, it contains only a few of the peptides similar to the peptides from the analyzed species. Therefore, this approach is an appropriate candidate for a decoy database.

**Fig. 5:**
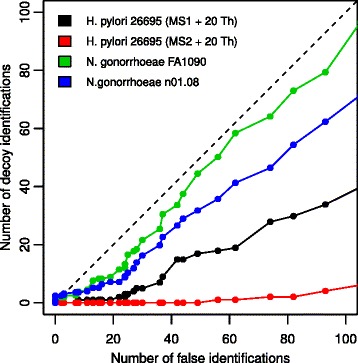
X-axis shows the number of false peptides identifications of *speptide* in the search the spectra of *H. pylori* H13-1 against the spectral library created from *H. pylori* 26695 spectra. False identifications assigned by additional Mascot search spectra of *H. pylori* H13-1 against protein database constructed from the annotated genome of *H. pylori* H13-1. Y-axis shows the number of peptides identifications of *H. pylori* H13-1 spectra in different *speptide* decoy searches: (i) shift of all MS1 peaks at +20 Th, (ii) shift of all MS2 peaks at +20 Th, (iii) library constructed from *N. gonorrhoeae* FA1090 spectra, (iv) library constructed from *N. gonorrhoeae* n01.08 spectra. The number of decoy identifications is normalized in a way that the number of spectrum-peptide pairs is the same in direct and decoy searches

Further we applied the SL created from *N. gonorrhoeae* MS/MS spectra to search for the SAPs in peptides among the spectra of *H. pylori* H13-1. Additionally, we have compared several approaches of peaks shifting to FDR estimation. Interestingly, using real spectra in our case appears to overestimate FDR slightly (Fig. 5), while artificial alterations of spectra underestimate FDR. The hypothesis is that the experimental spectra are more suitable for decoy database as by definition they correspond to initial database.

### Application of the method for strain-level bacterial differentiation

To validate the algorithm and demonstrate the features of the SAP identification method, we used MS/MS spectra for 73 *E. coli* isolates originating from the four different sources (raw sewage, and the freshly voided feces of feral cows, pet dogs and farm pigs)[15]. That work demonstrates the novel method to differentiate *E. coli* isolates by their animal sources based on similar spectra clusters between strains obtained by SpectraST [10].

In current study we show the possibility of differentiation *E. coli* strains by identified SAPs. All identified spectra without PTM (88,408 spectra and 5,618 unique peptides) were used to construct a spectral library containing ≤2 randomly selected spectra for each unique peptide (8,501 spectra). We performed a SAP search for the unidentified spectra against this library (5 % FDR). For each strain, the SAP-containing peptides were identified and combined into a substitution matrix. If either an original peptide or its variant with a SAP was not detected the sample, the peptide was assigned an NA value. As a result, a total of 98 unique peptides with SAPs were identified. Multi-dimensional scaling for spectral clusters and SAPs matricies for both approaches presented in supplementary (Additional file 1: Section 3.4).

**Fig. 6:**
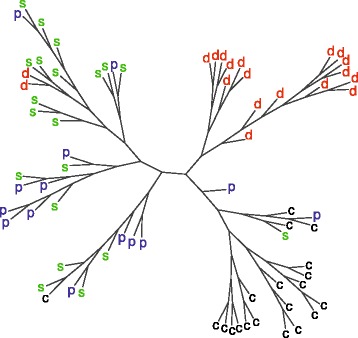
Unrooted phylogenetic tree of 73 *E. coli* isolates created with neighbor joining and additional maximum parsimony methods on the basis of identified SAPs, leaves coded by strains host organism: (p) - pig, (d) - dog, (c) - cow and (s) - raw sewage

Unlike the spectral cluster approach [15], the knowledge of amino acid polymorphisms allows to employ phylogenetic methods and reconstruct phylogenetic trees. For 73 *E. coli* isolates, we constructed a tree using NJ method with additional maximization of parsimony over the sites with available data on the presence or absence of a SAP in at least 80 % of the strains (Fig. 6).

The tree structure suggests the existence of 3 clusters: “dog”, “cow” and “sewage with pig” that corresponds to the results of [15]. It should be noted that, due to the unavailability of genome sequence data for 73 *E. coli* strains, we were unable to conduct the final verification of the identified SAPs, and the described validation procedure is based solely on the fact that the host-specific strains tend to form clusters corresponding to their hosts.

### Peptide bond study

In principle, the information about MS/MS spectrum alteration after SAP introduction could be used to identify the role of the amino acids in the fragmentation patterns. In the present study, the amount of the spectral pairs was over 19,000 and for some of the amino acid alterations, it was possible to collect over a hundred of unique spectra. We have calculated the relation between the angle and the SAP type and further performed a *t-test* to identify statistically significant differences between the different amino acid substitutions (Additional file 1: Section 4). Most of the pairs from the top of the list were different from those at the bottom (*p*≤1·10^−5^). This is in accordance with the observation that most similar amino acids like Ala and Gly have almost no effect on the spectrum as the spectral angle is high, while the substitutions like His and Asn have a low angle due to the difference in amino acids (hydrophobic and charged His is substituted by small Asn). We believe that the accumulation of the information would allow to construct a more precise algorithm and reveal the role of amino acids in peptide fragmentation.

## Conclusion

The developed method for spectral library-based identification of amino acid polymorphisms represents a perspective approach in the field of bacterial proteomics allowing to identify hundreds of peptides with novel SAPs from a single proteome with reliable estimation of the false discovery rate and removing unanticipated PTMs.

## Additional files

Additional file 1 Supplementary materials with **Figures S1-S12** and **Tables S1-S5**. (PDF 855 kb)

Additional file 2 Table with identifications of MS/MS spectra of *H. pylori* E48 and H-13 in classical Mascot search against sequence database and *speptide* search. (XLSX 89.6 kb)

## Abbreviations

FDR: False discovery rate
NJ: Neighbor joining
NA: Not available information peptide. PTM: Post-translational modification
ROC: Receiver operating characteristic
SNP: Single nucleotide polymorphism
SAP: Single amino acid polymorphism
