# Supplementary material for: Large scale analysis of amino acid substitutions in bacterial proteomics
Source: BMC Bioinformatics. 2016 Nov 8;17:450. doi: 10.1186/s12859-016-1301-5 (PMC5100282; doi:10.1186/s12859-016-1301-5)
Supplement: Supplementary file 1 — Supplementary materials with Figures S1-S12 and Tables S1-S5. (PDF 855 kb) [file 12859_2016_1301_MOESM1_ESM.pdf]

SUPPLEMENTARY MATERIALS

Large scale analysis of amino acid substitutions in bacterial proteomics

Dmitry Ischenko[1,2*], Dmitry Alexeev[1,2], Egor Shitikov[1], Alexandra Kanygina[1,2], Maja Malakhova[1], Elena Kostryukova[1], Andrey Larin[1], Sergey Kovalchuk[1], Olga Pobeguts[1], Ivan Butenko[1], Nikolay Anikanov[1], Ilya Altukhov[1,2], Elena Ilina[1] and Vadim Govorun[1]

[*]Correspondence:
dmitry.ischenko@phystech.edu
[1] Research Institute of Physical Chemical Medicine, Malaya Pirogovskaya, 1a, 119435 Moscow, Russian Federation
Full list of author information is available at the end of the article

Abstract

Background: Proteomics of bacterial pathogens is a developing field exploring microbial physiology, gene expression and the complex interactions between bacteria and their hosts. One of the complications in proteomic approach is micro- and macro-heterogeneity of bacterial species, which makes it impossible to build a comprehensive database of bacterial genomes for identification, while most of the existing algorithms rely largely on genomic data.

Results: Here we present a large scale study of identification of single amino acid polymorphisms between bacterial strains. An *ad hoc* method was developed based on MS/MS spectra comparison without the support of a genomic database. Whole-genome sequencing was used to validate the accuracy of polymorphism detection. Several approaches presented earlier to the proteomics community as useful for polymorphism detection were tested on isolates of *Helicobacter pylori*, *Neisseria gonorrhoeae* and *Escherichia coli*.

Conclusion: The developed method represents a perspective approach in the field of bacterial proteomics allowing to identify hundreds of peptides with novel SAPs from a single proteome.

Keywords: Spectral library; SAP

Contents

Abstract **1**

1 Standard procedures **3**
1.1 Protein database construction and spectra identification with Mascot 3
1.2 Sequence assignment and spectral library construction 3
1.3 Estimation of the number of polymorphisms based on genomic data 3

2 Algorithm **4**
2.1 Vector representation of spectra and angle calculation 4
2.2 Detection of identical spectra 5
2.2.1 Transformation methods 5
2.2.2 Parameters estimation (N, I, $\cos\theta$) 5
2.3 SAP detection algorithm 7
2.3.1 Choosing a set of possible SAPs 7
2.3.2 Peaks annotation (b-, y- and additional ion series) 8
2.3.3 Different methods of peaks shifting 9
2.3.4 Parameters estimation (C, I, alg, $\cos\theta$) 9

3 Algorithm exploration **12**
3.1 NA exploration 12
3.2 Decoy database research 12
3.3 Comparison with other algorithms (Byonic, pMatch, SPIDER) . . . 14
3.4 73 *E.coli* differentiation study 15
3.5 Detection of point mutations 17

4 SNP study **18**
4.1 Angle dependence on the type of SAP 18

5 Software and data **19**
5.1 Software and programming languages 19
5.2 Data and code availability 19

6 References **20**

1 Standard procedures

1.1 Protein database construction and spectra identification with Mascot

Raw data files with WIFF and .D file format were converted to the Mascot generic format (MGF file format) using AB SCIEX MS Data Converter version 1.3 and Compass Data Analysis 4.2 (Build 383.1) respectively. Mascot 2.2.07 was used for the identification with the following parameters: MS1 tol: 10 ppm, MS2 tol: 0.5 Da, varibale modifications: Oxidation(M) and Carbomidomethylation(C), trypsin specificity with 1 missed cleavages allowed. Decoy searches were implemented by database construction with reversed proteins.

1.2 Sequence assignment and spectral library construction

The spectral libraries were constructed from Mascot identification results excluding those with predicted post-translational modifications. The threshold values were estimated for $FDR \leq 0.05$. Each spectral library represents a file in MGF format with an additional SEQ field, which contains an amino acid sequence of the peptide identified. The resulting library sizes for each strain are shown in Table S-2:

Table S-1 Spectral library sizes.

Strain	NCBI accession	Spectral library size
H. pylori A45	AMYU00000000	13657
H. pylori 26695	NC_018939	13306
H. pylori J99	NC_000921	13789
H. pylori E48	AYHQ00000000	12440
H. pylori H13-1	AYUH00000000	15194
N. gonorrhoeae i19.05	JFBA00000000	29462
N. gonorrhoeae n01.08	JIBZ00000000	32815
N. gonorrhoeae FA1090	NC_002946	10854

To construct a spectral library we used an MGF format file containing all the data of a proteomic experiment and Mascot result export file (described above). We used the *speptide* software to construct the library. The filtering according to peptide identification criteria was applied. The results of spectra identification using Mascot should be exported in CSV format file containing "Query title" and "Sequence" fields (pep_scan_title and pep_seq). After the spectral library was annotated, we match ion series to MS/MS peaks using the amino acid sequence of peptides identified by Mascot. Spectra for the known genome were identified with Mascot using the genome of known organism and further used as spectral library. MS/MS peaks in the spectral library are annotated and ion series are assigned.

1.3 Estimation of the number of polymorphisms based on genomic data

The number of SAPs in tryptic peptides between a pair of strains was estimated using *blastp* package. For each of the comparison pair of datasets the lists with all tryptic peptides were recieved with *ad hoc* perl scripts (Fig. S-1). The database for *blastp* search was created from the list of peptides sequences from the first sample.

The list of sequences of the second sample were searched against created database. At the last step only peptides with the same length and differ only in one amino acid were involved in the estimation of the total number of possible SAPs identification.

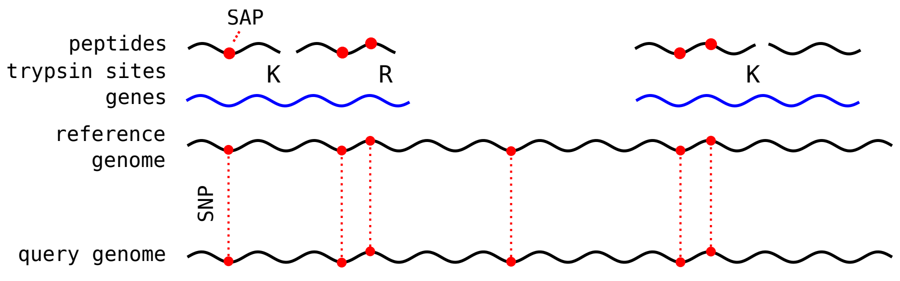

Figure S-1 Estimation of the number of SAPs in peptides based on genomes information.

2 Algorithm

2.1 Vector representation of spectra and angle calculation

The comparison of the spectra is based on spectral angle calculation described earlier[1, 2]. A spectrum is represented by a vector in a space m with components corresponding to the intensities of the peaks of this spectrum. The estimation of similarity between two spectra is based on the calculation of the cosine similarity between the corresponding vectors.

Briefly, every spectrum is represented by two ordered sets $\{m\}$ and $\{i\}$, containing $\frac{m}{z}$ and intensities of peaks, correspondingly, and function $I_s : \{m\} \to \{i\}$, putting in accordance $\frac{m}{z}$ and its intensity. Further two symbols are used for brevity: first $\in^\delta$ ("belongs to with accuracy of δ"),

$$x \in^\delta \{M\} \Leftrightarrow \exists m \in \{M\} : |x - m| < \delta$$

Second: $\cup^\delta$ ("unity with accuracy of δ"),

$$A \cup^\delta B = A' \cup B, A' = \{a\} : a \notin^\delta B$$

Comparison of the two spectra S_1 ($\{m_1\}$, $\{i_1\}$, I_{s_1}) and S_2 ($\{m_2\}$, $\{i_2\}$, I_{s_2}) is based on contruction of a set M $= \{m_1\} \cup^\delta \{m_2\}$. For every spectrum in the set M, we define a function $I_s^\delta(x) : M \to \{i\}$:

$$I_{s_n}^\delta(x) = \begin{cases} I_{S_n}(m), & \text{if } \exists m \in \{m_n\} : |x - m| < \delta. \\ 0, & \text{otherwise.} \end{cases}$$

Thus, we associate the spectra compared with intensity ordered sets (vectors) $I_{s_1}^\delta(M)$ and $I_{s_2}^\delta(M)$, and as measure of similarity for the two spectra, we use the

angle value between those two vectors $\cos\Theta = \cos(I^{\delta}_{s_1}(M), I^{\delta}_{s_2}(M))$. The higher is the cosine of the angle value, the higher is the probability that the spectra correspond to similar sequences.

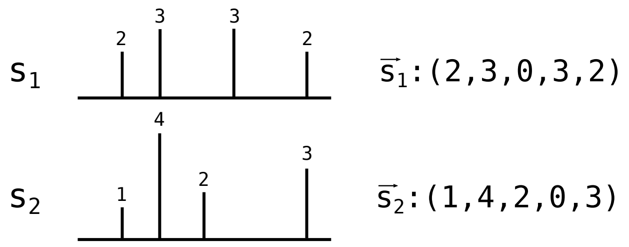

Figure S-2 Representation of spectrum as a vector.

$$\cos\Theta = \frac{\vec{s_1}\cdot\vec{s_2}}{|s_1|\cdot|s_2|}$$

2.2 Detection of identical spectra

For two spectral sets (SP and SL) with a given Δ_{MS1} candidate pairs of spectra are defined as those with $|MS1_{SP} - MS1_{SL}| \leq \Delta_{MS1}$. Each spectrum in a pair is represented by a vector, and the cosine similarity is calculated. Each spectrum from SP is assigned to a spectrum from SL corresponding to the minimal angle between two vectors.

2.2.1 Transformation methods

The initial spectra is not always useful for calculation of spectral angle. Several approaches exists to transform the initial spectra[3]. It is known that the transformation of intensity improves the results. It was also shown that limiting the number of peaks taken into comparison can improve the comparison. For convenience the spectral intensity is normalized so that the average intensity in the spectra equals 100. The following several intensity transformations are applied: $\sqrt{I}$, $\ln I$ and untransformed intensity value I.

2.2.2 Parameters estimation (N, I, $\cos\theta$)

We used spectral sets from three strains of *H. pylori*: A45, J99 and 26695 as a training set for the algorithm. Three pairwise comparisons are performed; in each of them, one sample is considered as a reference sample and the other as a query sample. A45 $\rightarrow$ J99, J99 $\rightarrow$ 26695, 26695 $\rightarrow$ A45 (the second sample in each pair serving as reference). Different methods of intensity transformations are applied; the number of the peaks selected for further analysis is also varied. $\frac{m}{N}$ most intensive peaks are selected, where m is MS1 mass and N – is a parameter varying from 10 to 200. The results for each strain are verified by comparison with Mascot identifications using the protein database built from the annotation of the

corresponding genome. For each parameter values areas under ROC curves are calculated, and the number of true positive identifications is estimated for FDR not exceeding 0.05. (Table S-4).

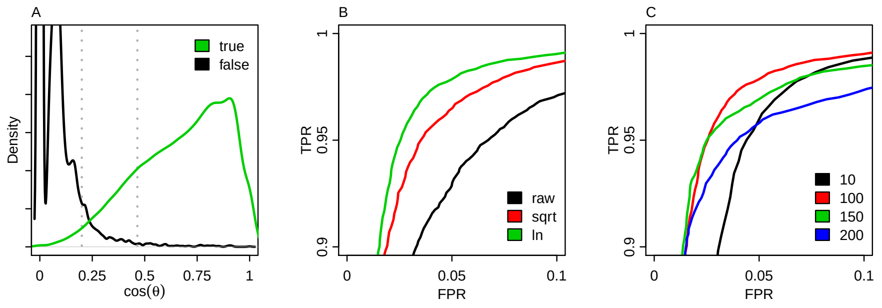

Figure S-3 **A**. Probability density of $\cos\Theta$ between the spectra, corresponding to similar and different peptide sequences ($\ln I$ transformation, top $\frac{m}{100}$ peaks). **B**. ROC curves for different methods of intensity transformation. **C**. ROC curves for different values of N (top $\frac{m}{N}$ peaks).

The resulting parameter and threshold $\cos\Theta$ values are defined for 0.05 and 0.01 FDR: $\ln I$ transformation, $N = 100$ (top $\frac{m}{N}$ peaks), $\cos\Theta >= 0.31$ (0.47).

Table S-2 Number of identified peptides with $FDR \leq 0.05$ and area under ROC-curves for different parameters for algorithm of identical spectra identification.

	Number of peptides			Area under ROC-curve		
N **(top $\frac{m}{n}$ peaks)**	$\sqrt{I}$	$\ln I$	I	$\sqrt{I}$	$\ln I$	I
10	4210	4210	4125	0.987	0.986	0.981
20	4210	4215	4122	0.988	0.986	0.981
30	4204	4210	4115	0.988	0.987	0.981
40	4197	4213	4113	0.988	0.988	0.981
50	4187	4206	4112	0.989	0.989	0.982
60	4186	4210	4116	0.989	0.990	0.983
70	4182	4214	4111	0.990	0.991	0.984
80	4179	4209	4097	0.991	0.992	0.985
90	4166	4199	4101	0.991	0.992	0.985
100	4158	4198	4095	0.991	0.992	0.986
110	4149	4196	4091	0.991	0.992	0.986
120	4140	4191	4086	0.990	0.991	0.986
130	4133	4179	4084	0.990	0.991	0.985
140	4132	4177	4078	0.989	0.991	0.985
150	4126	4171	4069	0.989	0.990	0.985
160	4110	4169	4054	0.988	0.989	0.984
170	4106	4158	4055	0.987	0.988	0.984
180	4092	4144	4047	0.986	0.987	0.983
190	4088	4137	4038	0.985	0.986	0.982
200	4082	4130	4031	0.984	0.985	0.981

The resulting parameter and threshold $\cos\Theta$ values are defined for 0.05 and 0.01 FDR.

2.3 SAP detection algorithm

For two spectral sets (SP and SL) with a given Δ_{MS1} candidate pairs of spectra are defined as those with $\exists D_\delta \in \{D\} : D_\delta - \Delta_{MS1} \leq MS1_{SL} - MS1_{SP} \leq D_\delta + \Delta_{MS1}$, where $\{D\}$ is a set of mass differences for selected amino acid substitutions. A spectrum from SL is transformed according to the chosen method. Each spectrum in a pair is represented by a vector, and the cosine similarity is calculated. Each spectrum from SP is assigned to a spectrum from SL corresponding to the minimal angle between two vectors.

2.3.1 Choosing a set of possible SAPs

Only amino acid substitutions caused by a single nucleotide change in a codon were selected for the analysis; substitutions with $\Delta_m \leq 1Da$ were excluded, resulting in total of 138 substitutions under consideration.

Table S-3 SAPs selected for the analysis (the absolute value of Δ_m is specified).

K ↔ M	2.9455	N ↔ H	23.0160	L ↔ R	43.0170
P ↔ T	3.9949	L ↔ H	23.9748	I ↔ R	43.0170
Q ↔ H	9.0003	M ↔ R	25.0606	A ↔ D	43.9898
S ↔ P	10.0207	H ↔ Y	26.0044	C ↔ F	44.0592
T ↔ I	12.0364	A ↔ P	26.0157	G ↔ C	45.9877
T ↔ N	12.9952	S ↔ L	26.0520	V ↔ F	48.0000
V ↔ I	14.0157	S ↔ I	26.0520	D ↔ Y	48.0364
V ↔ L	14.0157	S ↔ N	27.0109	N ↔ Y	49.0204
D ↔ E	14.0157	T ↔ K	27.0473	C ↔ R	53.0919
G ↔ A	14.0157	K ↔ R	28.0061	T ↔ R	55.0534
S ↔ T	14.0157	A ↔ V	28.0313	A ↔ E	58.0055
N ↔ K	14.0520	Q ↔ R	28.0425	G ↔ D	58.0055
L ↔ Q	14.9745	V ↔ E	29.9742	P ↔ R	59.0483
I ↔ K	15.0109	R ↔ W	29.9782	S ↔ F	60.0364
V ↔ D	15.9585	T ↔ M	29.9928	C ↔ Y	60.0541
S ↔ C	15.9772	G ↔ S	30.0106	S ↔ R	69.0691
F ↔ Y	15.9949	A ↔ T	30.0106	G ↔ E	72.0211
A ↔ S	15.9949	P ↔ Q	31.0058	L ↔ W	72.9952
P ↔ L	16.0313	V ↔ M	31.9721	S ↔ Y	76.0313
L ↔ M	17.9564	L ↔ F	33.9844	C ↔ W	83.0701
I ↔ M	17.9564	I ↔ F	33.9844	S ↔ W	99.0473
H ↔ R	19.0422	P ↔ H	40.0061	G ↔ R	99.0796
D ↔ H	22.0320	G ↔ V	42.0470	G ↔ W	129.0578

2.3.2 Peaks annotation (b-, y- and additional ion series)

For SAP identification, peaks in a spectral library are annotated. Their intersection with theoretical peaks of peptide sequence with a given Δ ($\Delta = 0.5\ Da$ in current study) is constructed. Different ion types were considered, $b-, y-, b-H_20-, b-NH_3-, y-H_2O-, y-NH_3-$ ions in particular. The best algorithm was selected after using different types of ions for training. After comparing the results of identifications using different approaches, only $b-, y-$ ions were selected for the analysis, while the others were considered as ”unannotated”.

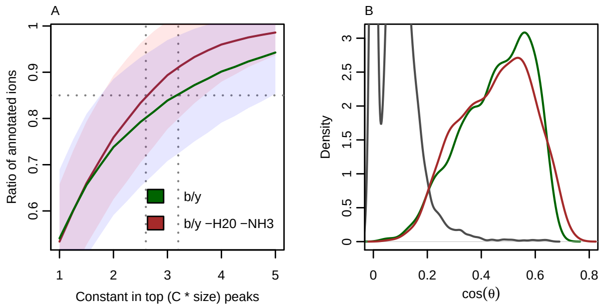

Figure S-4 A. Fraction of all annotated peaks depending on the selected top intensity peaks for different ion series. **B**. Probability density of $\cos\Theta$ for true and false SAP identifications for different ion series. **C**. Distributions of $\log$ intensities for different ion series.

2.3.3 Different methods of peaks shifting

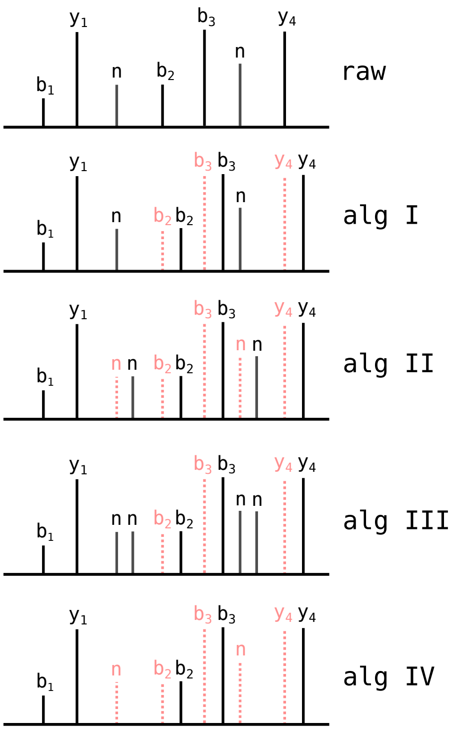

Figure S-5 **alg I**. All annotated peaks containing the respective substitution are shifted, all unannotated peaks remain in place. First $\frac{m}{N}$ most intensive peaks from SP and SL spectra are used. **alg II**. All annotated peaks containing the respective substitution are shifted by, all unannotated peaks are also shifted. First $\frac{m}{N}$ most intensive peaks from SP and SL spectra are used. **alg III**. All annotated peaks containing the respective substitution are shifted, all unannotated peaks remain in place and additional set of peaks is added corresponding to shifted unannotated peaks. First $\frac{m}{N}$ most intensive peaks from SP and SL are used. **alg IV**. All annotated peaks containing the respective substitution are shifted by, all unannotated peaks are removed from SL spectra. All the annotated ions from SL are used, first $C \cdot S$ most intensive peaks from SP are used.

2.3.4 Parameters estimation (C, I, alg, $\cos\theta$)

We used spectral sets from three strains of *H. pylori*: A45, J99 and 26695, as a training dataset for the algorithm. Three pairwise comparisons are performed; in each of them, one sample is considered as a reference sample and the other as a query sample. A45 $\rightarrow$ J99, J99 $\rightarrow$ 26695, 26695 $\rightarrow$ A45 (the second sample in each pair serving as reference). Different methods of shifting of the peaks in the reference spectrum and intensity transformation are applied; the number of the peaks from reference and query spectra selected for further analysis is also varied. In the reference spectrum, $\frac{m}{N}$ most intensive peaks are selected, where m is MS1 mass and N – is a parameter varying from 10 to 200. The selected peaks are then annotated and shifted according to one of the methods. In the query spectrum, $C \cdot S$ most intensive peaks are selected, where S is the number of peaks in the reference spectrum left after filtering and annotation and C is a parameter varying from 1 to

5. The results for each strain are verified by comparison with Mascot identifications using the protein database built from the annotation of the corresponding genome. For each parameter values areas under ROC curves are calculated, and the number of true positive identifications is estimated for FDR not exceeding 0.05 (Table S-6).

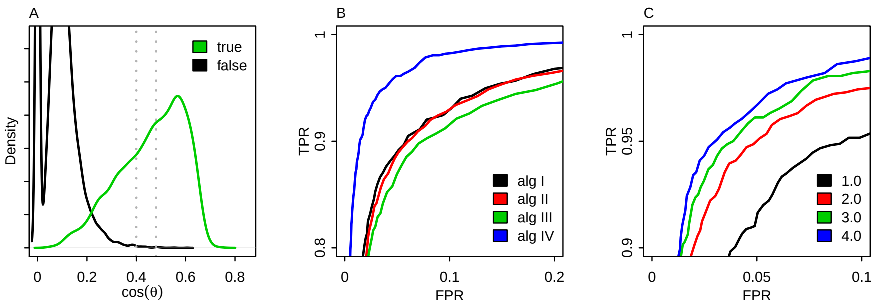

Figure S-6 **A**. Probability density of $\cos\Theta$ between the spectra, corresponding to true SAP and random match (alg IV, $\ln I$ intensity transformation, top $3.2 \cdot S$ peaks). **B**. ROC curves for different methods of peaks shifting in reference spectrum. **C**. ROC curves for different values of C (top $C \cdot S$ peaks).

The resulting parameter and threshold $\cos\Theta$ values are defined for 0.05 and 0.01 FDR: alg IV, $\ln I$ transformation, $C = 3.2$ (top $C \cdot S$ peaks), $\cos\Theta >= 0.40$ (0.48)

Table S-4 Number of identified peptides with $FDR \leq 0.05$ and areas under ROC-curves for different parameters for algorithm of SAP identification.

alg	alg I									alg II									alg III									alg IV		
$trans$	$\sqrt{I}$			$\ln I$			I			$\sqrt{I}$			$\ln I$			I			$\sqrt{I}$			$\ln I$			I			$\sqrt{I}$	$\ln I$	I
N	100	150	50	100	150	50	100	150	50	100	150	50	100	150	50	100	150	50	100	150	50	100	150	50	100	150	50	1	1	1
	Number of peptides																													
C																														
1.0	861	767	909	943	818	965	594	527	605	751	644	873	869	794	893	412	400	415	790	714	872	880	817	816	516	448	539	1012	1045	574
1.2	853	794	909	934	816	956	602	570	609	763	640	886	850	756	864	415	401	416	796	747	858	887	806	827	526	425	549	1042	1089	590
1.4	861	802	891	946	844	939	586	534	605	775	630	860	788	714	881	416	413	413	790	729	851	851	804	855	554	462	545	1043	1113	590
1.6	835	823	883	898	878	942	591	534	602	785	648	877	799	732	854	408	415	410	805	739	870	819	818	862	533	462	545	1037	1119	575
1.8	830	796	870	910	898	957	599	538	597	796	634	860	812	689	860	414	414	409	795	741	885	797	843	856	550	457	544	1050	1138	564
2.0	822	808	884	933	907	951	591	535	593	767	647	870	818	723	837	404	413	406	782	734	884	801	861	852	543	462	543	1077	1161	579
2.2	835	807	888	952	928	920	585	533	589	742	657	873	800	727	844	399	407	404	788	730	881	807	834	869	539	456	543	1087	1176	585
2.4	844	803	894	936	933	910	576	536	588	741	665	881	802	744	826	404	413	404	789	734	894	801	855	868	537	462	543	1088	1184	576
2.6	856	805	890	944	926	933	585	541	584	717	687	876	791	762	862	403	409	412	790	733	893	805	824	867	535	481	543	1107	1200	569
2.8	857	804	901	904	918	924	579	542	584	752	683	872	790	766	852	402	412	412	762	732	877	799	782	867	518	473	543	1098	1202	569
3.0	837	783	897	885	906	923	578	542	598	751	674	870	789	789	849	402	408	412	781	742	877	831	790	866	515	483	543	1098	1213	571
3.2	818	780	917	889	915	926	583	541	598	741	691	867	787	779	844	405	407	411	794	729	878	844	793	866	514	478	543	1106	1216	557
3.4	822	783	932	890	886	920	583	531	598	746	688	867	783	790	839	404	403	411	791	711	877	856	787	866	514	476	543	1096	1221	552
3.6	828	783	931	891	879	920	573	538	598	735	628	867	784	767	837	404	388	411	788	712	877	847	808	866	514	471	543	1104	1215	562
3.8	833	784	931	868	877	920	578	535	598	725	627	866	743	763	835	403	393	411	802	716	877	842	770	866	536	470	543	1099	1220	559
4.0	838	785	931	869	851	920	577	540	598	716	608	866	725	738	833	402	390	411	813	719	877	788	756	866	533	468	543	1101	1239	555
4.2	835	787	930	881	851	919	576	537	598	713	614	866	700	739	833	401	387	411	812	712	877	834	735	866	533	463	543	1125	1237	551
4.4	842	778	929	892	875	918	574	533	598	705	591	866	712	739	833	399	385	411	811	720	877	829	745	866	533	461	543	1128	1232	562
4.6	852	782	930	883	855	918	574	532	598	699	592	866	700	740	833	399	394	411	811	726	877	828	759	866	533	460	543	1131	1237	562
4.8	849	774	930	877	822	919	573	532	598	695	580	866	693	723	833	398	392	411	811	722	877	827	771	866	533	460	543	1126	1245	575
5.0	857	777	930	869	827	919	572	529	598	690	604	866	707	728	833	397	391	411	824	717	877	825	757	866	533	475	543	1120	1242	545
	Area under ROC-curve																													
1.0	0.930	0.898	0.923	0.904	0.939	0.898	0.905	0.899	0.916	0.903	0.895	0.884	0.939	0.885	0.940	0.874	0.878	0.883	0.893	0.915	0.884	0.871	0.904	0.860	0.902	0.864	0.890	0.948	0.936	0.933
1.2	0.933	0.889	0.921	0.910	0.933	0.902	0.916	0.875	0.918	0.909	0.888	0.895	0.878	0.876	0.862	0.887	0.891	0.891	0.909	0.914	0.894	0.880	0.906	0.862	0.858	0.873	0.893	0.939	0.931	0.912
1.4	0.932	0.929	0.933	0.908	0.908	0.905	0.916	0.909	0.917	0.906	0.922	0.901	0.947	0.908	0.865	0.888	0.895	0.894	0.899	0.915	0.898	0.876	0.905	0.861	0.861	0.875	0.895	0.931	0.965	0.921
1.6	0.933	0.921	0.935	0.912	0.894	0.907	0.872	0.880	0.923	0.908	0.918	0.908	0.876	0.888	0.872	0.890	0.856	0.899	0.900	0.921	0.899	0.867	0.902	0.861	0.866	0.876	0.898	0.966	0.936	0.925
1.8	0.934	0.918	0.938	0.919	0.890	0.907	0.875	0.889	0.884	0.912	0.914	0.907	0.883	0.877	0.869	0.889	0.863	0.903	0.900	0.928	0.902	0.864	0.908	0.861	0.872	0.875	0.899	0.960	0.923	0.931
2.0	0.932	0.909	0.939	0.914	0.937	0.894	0.878	0.890	0.885	0.910	0.899	0.910	0.881	0.926	0.866	0.892	0.862	0.903	0.898	0.928	0.901	0.853	0.899	0.860	0.873	0.880	0.899	0.948	0.956	0.932
2.2	0.929	0.903	0.940	0.905	0.931	0.893	0.877	0.889	0.885	0.914	0.892	0.910	0.882	0.920	0.872	0.891	0.862	0.905	0.901	0.928	0.902	0.937	0.893	0.861	0.873	0.879	0.899	0.948	0.948	0.934
2.4	0.927	0.900	0.942	0.893	0.932	0.897	0.875	0.889	0.888	0.912	0.889	0.913	0.880	0.926	0.873	0.894	0.865	0.905	0.899	0.921	0.902	0.930	0.881	0.859	0.873	0.880	0.900	0.941	0.940	0.934
2.6	0.929	0.899	0.943	0.886	0.925	0.891	0.875	0.888	0.887	0.907	0.882	0.918	0.866	0.917	0.873	0.895	0.867	0.906	0.896	0.917	0.903	0.925	0.873	0.860	0.875	0.880	0.900	0.936	0.929	0.933
2.8	0.927	0.900	0.942	0.883	0.926	0.895	0.876	0.889	0.888	0.905	0.880	0.917	0.862	0.928	0.877	0.897	0.868	0.907	0.894	0.913	0.904	0.925	0.869	0.860	0.876	0.879	0.900	0.931	0.913	0.935
3.0	0.924	0.898	0.941	0.876	0.922	0.894	0.878	0.889	0.888	0.899	0.878	0.921	0.856	0.914	0.877	0.900	0.869	0.907	0.891	0.911	0.904	0.924	0.864	0.860	0.875	0.879	0.900	0.930	0.981	0.937
3.2	0.921	0.896	0.941	0.945	0.920	0.891	0.879	0.890	0.887	0.898	0.881	0.920	0.946	0.903	0.877	0.900	0.869	0.907	0.894	0.909	0.903	0.923	0.862	0.859	0.876	0.878	0.900	0.932	0.982	0.937
3.4	0.917	0.894	0.941	0.936	0.914	0.890	0.881	0.890	0.888	0.895	0.878	0.919	0.849	0.898	0.875	0.897	0.871	0.907	0.891	0.906	0.903	0.921	0.856	0.859	0.876	0.878	0.900	0.930	0.969	0.935
3.6	0.921	0.891	0.941	0.934	0.910	0.891	0.881	0.887	0.888	0.894	0.877	0.919	0.945	0.896	0.875	0.898	0.871	0.907	0.889	0.903	0.903	0.921	0.931	0.859	0.876	0.878	0.900	0.929	0.967	0.936
3.8	0.918	0.887	0.941	0.930	0.902	0.890	0.880	0.887	0.888	0.898	0.874	0.920	0.941	0.888	0.876	0.900	0.870	0.907	0.888	0.899	0.903	0.921	0.928	0.859	0.876	0.879	0.900	0.926	0.960	0.935
4.0	0.914	0.882	0.942	0.925	0.902	0.889	0.879	0.887	0.888	0.898	0.872	0.921	0.939	0.893	0.875	0.903	0.868	0.907	0.888	0.900	0.903	0.921	0.924	0.859	0.876	0.879	0.900	0.925	0.957	0.934
4.2	0.912	0.882	0.942	0.920	0.901	0.890	0.878	0.887	0.888	0.896	0.871	0.921	0.942	0.889	0.877	0.902	0.868	0.907	0.889	0.898	0.903	0.921	0.921	0.859	0.877	0.878	0.900	0.921	0.952	0.935
4.4	0.910	0.938	0.941	0.920	0.896	0.890	0.877	0.888	0.888	0.894	0.871	0.920	0.941	0.885	0.875	0.901	0.869	0.908	0.889	0.896	0.903	0.919	0.920	0.859	0.877	0.877	0.900	0.977	0.945	0.935
4.6	0.908	0.936	0.942	0.922	0.893	0.889	0.878	0.889	0.888	0.896	0.870	0.921	0.939	0.882	0.875	0.902	0.868	0.908	0.889	0.898	0.903	0.920	0.914	0.859	0.877	0.878	0.900	0.976	0.944	0.934
4.8	0.910	0.931	0.942	0.924	0.891	0.889	0.878	0.885	0.888	0.897	0.866	0.921	0.941	0.879	0.875	0.901	0.870	0.908	0.889	0.897	0.903	0.920	0.912	0.859	0.877	0.878	0.900	0.917	0.943	0.934
5.0	0.910	0.930	0.942	0.922	0.887	0.889	0.878	0.883	0.888	0.897	0.933	0.921	0.939	0.880	0.875	0.901	0.870	0.908	0.887	0.897	0.903	0.920	0.911	0.859	0.877	0.879	0.900	0.917	0.945	0.933

3 Algorithm exploration

3.1 NA exploration

Spectra identified with *speptide* but not identified with Mascot using genome-derived database are called NA (information not available) spectra. Indirect verification of the identification results included search for homology between peptides in reference and query strains using *blast* and estimation of the number of SAPs in homologous peptides. Only those reference peptides were used for the alignment, for which SAPs in query peptides were identified but not confirmed with Mascot. In each search, a genome-derived blast database was constructed using *makeblastdb* tool and the reference peptides were then aligned with *tblastn* (60% identity) against this database. Hamming distance was employed to estimate the number of amino acid variations in homologous peptides. The results are shown in Fig. S-7.

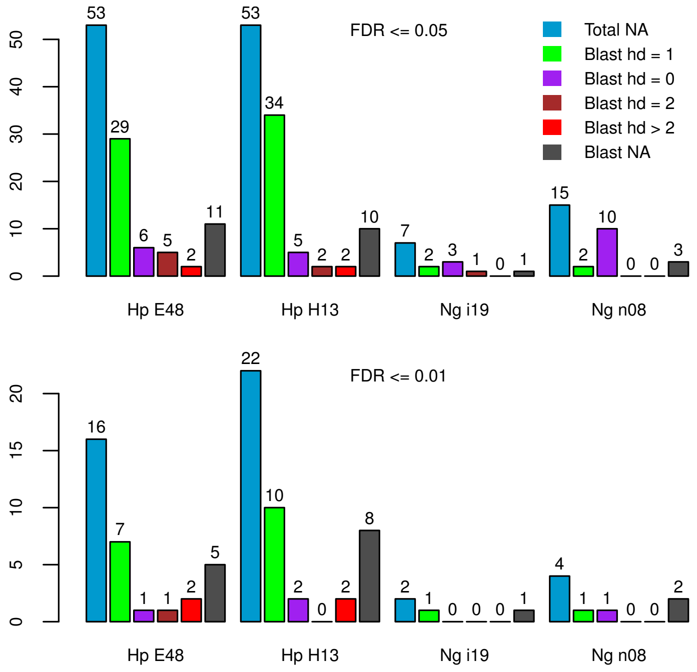

Figure S-7 Analysis of NA identifications by aligning using *tblastn* reference peptides against own genomes. Colors encode different distance between the reference and query peptides (one SAP encoded by green color, $hd = 1$).

3.2 Decoy database research

With a purpose to introduce an additional FDR estimation we explored different ways of constructing decoy spectral librarires. We used well-known approaches: shift $\frac{m}{z}$ values for all MS1 peaks [4] (+20 Th), shift $\frac{m}{z}$ values of all MS2 peaks in each spectrum (+20 Th)[5]. In addition, we have considered constructing decoy spectral library database from the spectra of different bacterial species, putting them under one of the following additional conditions: (1) equal number of peptides in the decoy and direct spectral libraries or (2) equal number of of spectrum-peptide pairs

(difference in MS1 m values falling into the set of amino acid deltas) for direct and decoy searches. The results are presented at Fig. S-8:

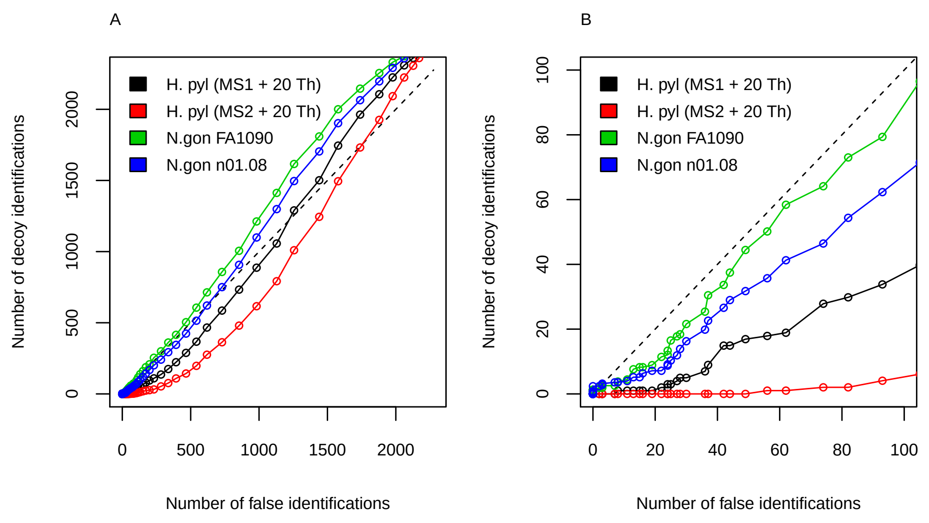

Figure S-8 X-axis shows the number of false peptides identifications of *speptide* in the search the spectra of *H. pylori* H13-1 against the spectral library created from *H. pylori* 26695 spectra. False identifications assigned by additional Mascot search spectra of *H. pylori* H13-1 against protein database constructed from the annotated genome of *H. pylori* H13-1. Y-axis shows the number of peptides identifications of *H. pylori* H13-1 spectra in different *speptide* decoy searches: (i) shift of all MS1 peaks at +20 Th, (ii) shift of all MS2 peaks at +20 Th, (iii) library constructed from *N. gonorrhoeae* FA1090 spectra, (iv) library constructed from *N. gonorrhoeae* n01.08 spectra. The number of decoy identifications is normalized in a way that the number of spectrum-peptide pairs is the same in direct and decoy searches. Figures **A** and **B** represent different axis ranges. **A**. Axis range: $[0;\ 1500]$. **B**. Axis range: $[0;\ 100]$ (FDR $\in [0;\ 0.1]$).

3.3 Comparison with other algorithms (Byonic, pMatch, SPIDER)

Speptide was compared with three packages: Byonic[7], pMatch[8] and SPIDER[9]. Two of them (Byonic and pMatch) are not designed for direct search of amino acid substitutions, so their results required some post-processing in order to choose only those identifications that could be a result of an amino acid substitution according to their Δ_{MS1}. *H. pylori* E48 spectra were used as a query sample. The triple *H. pylori* database (A45, J99, 26695) including all identified peptides was used as a peptide database for *speptide*. The results from all four algorithms were compared with *H. pylori* E48 spectra genome-based identification with Mascot. The running parameters are listed below:

Byonic v.2.5.6. Precursor tolerance: 10 ppm, Fragment tolerance: 0.5 Da, Cleavage site(s): RK, Digestion specificity: Fully specific, Wildcard search, Total common max: 1, Minimum mass: -130, Maximum mass: 130. **pMatch v.1.5.0.1**. Precursor Tolerance: 20, M/Z Tolerance: 0.5, Shift Threshold: 3.0, Theta: 0.2. **PTM search + SPIDER (PeakStudio v7.0)**. Parent Mass Tolerance: 10 ppm, Maximum allowed PTM: 1, Fragment ion tolerance: 0.5 Da, De novo socore greater than: 50%, Peptide score less than: 15.

The results were filtered in order to fit $FDR \leq 0.05$.

Table S-5 Time usage for different algorithms (CPU 2.13GHz, 8Gb RAM). *H. pylori* E48 ($64 \cdot 10^3$ spectra) vs *H. pylori* 3 strain database ($40 \cdot 10^3$ spectra, $11 \cdot 10^3$ peptides)

	Speptide	pMatch	Byonic	SPIDER
CPU time:	∼10 min	∼5 min	∼3 hrs	∼1 hr

3.4 73 *E.coli* differentiation study

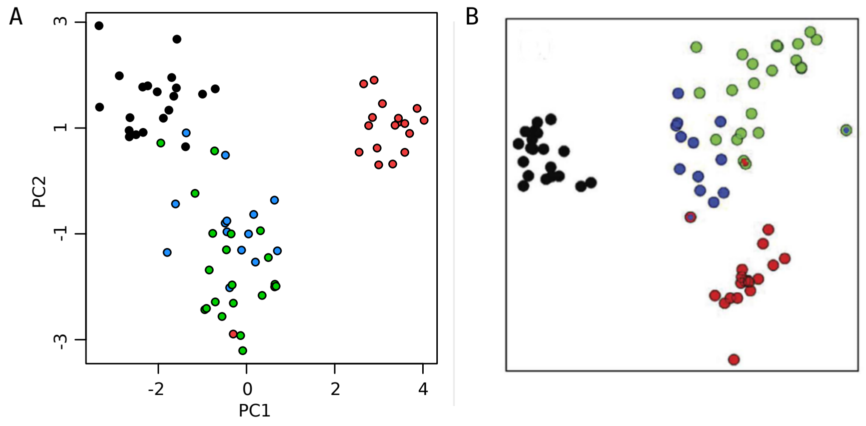

Figure S-9 Multi-dimensional scaling of 73 *E. coli* isolates on the basis of **A**. Identified SAPs **B**. UNID-proteomic[10]. Each dot represents a different isolate. Dots in green, black, red and blue represent isolates of sewage, cow, dog and pig, respectively.

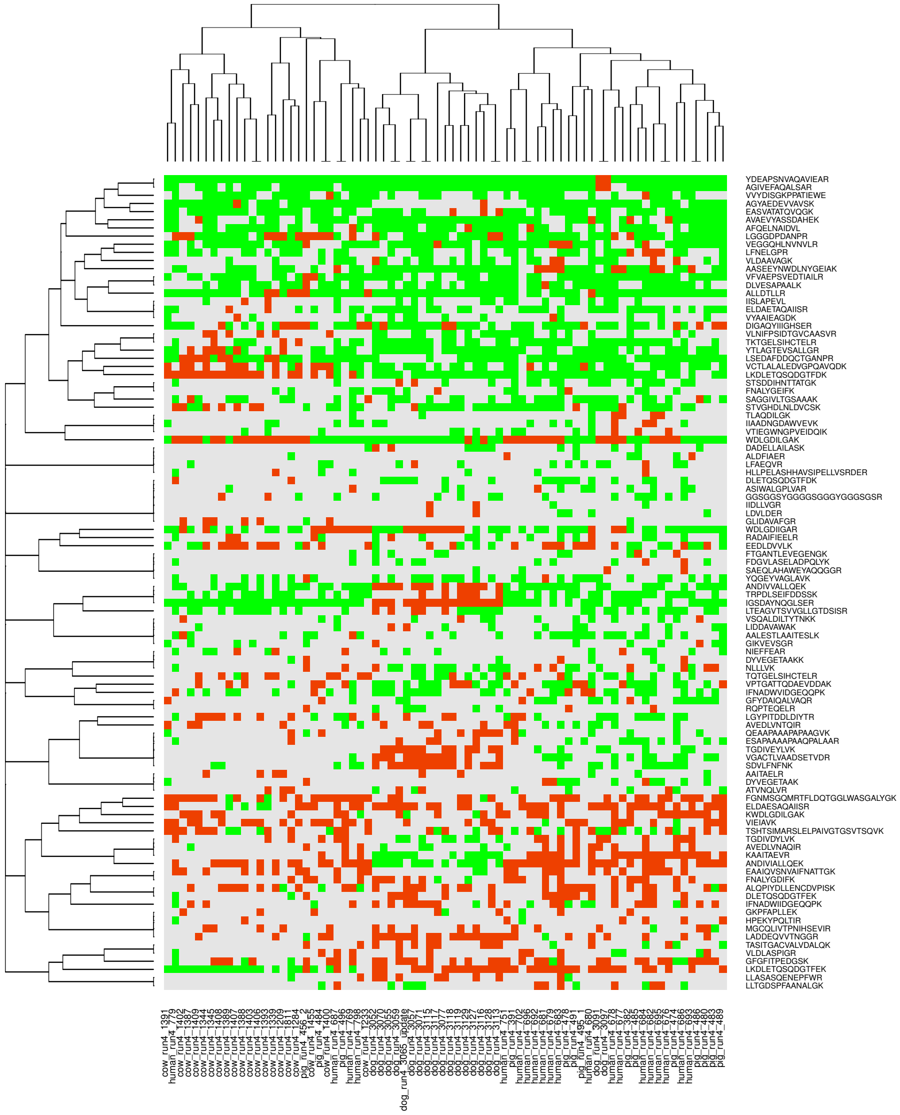

Figure S-10 Heatmap for identified peptides with SAP in 73 *E. coli* strains. Color encodes one of three states of peptide in a sample: green - original peptide, red - peptide with SAP, gray - the absence of both variants in a sample.

3.5 Detection of point mutations

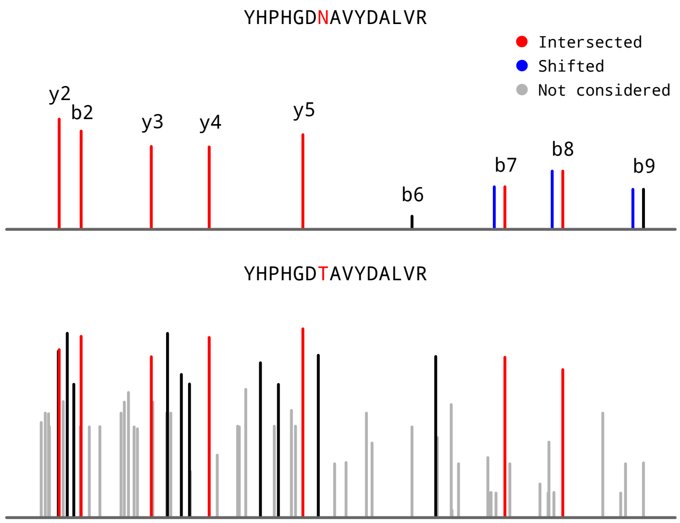

Figure S-11 Demonstration of identification possibility in QRDR region of gyrA gene of *H. pylori*. Above is the spectrum from spectral library with N in position 87 of a protein, below experimental spectrum with T in position 87 of a protein. Peaks that are used for identification are denoted as intersected, the b7 and b8 peaks containing the mass shifted ions with amino acid substitutions are identified.

4 SNP study

4.1 Angle dependence on the type of SAP

We calculated the angle dependence on the SAP type and further performed a *t-test* to identify statistically significant differences between the different amino acid substitutions. Only amino acid substitutions with at least 100 spectral pairs assigned were selected. Final p-values were adjusted with Benjamini-Hochberg correction.

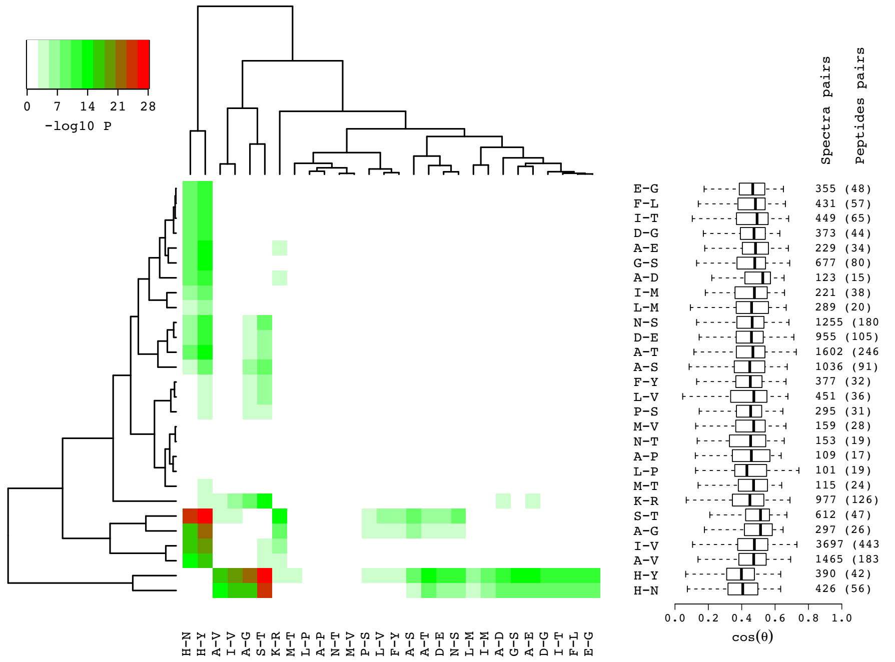

Figure S-12 Heatmap with $-\log_{10}$ Benjamini-Hochberg corrected p-values from *t-test* for $\cos\Theta$ for each pair of SAPs.

5 Software and data

5.1 Software and programming languages

- AB SCIEX MS Data Converter version 1.3 and Compass Data Analysis 4.2 : spectra conversion.
- Newbler 2.6 : read assembly.
- Mascot 2.2.07 : spectra identification.
- Blast 2.2.30 : peptides homology search and estimation of possible number of SAPs detection.
- Artemis (Version 16) : ORF search in genomes.
- R 3.2.1 : *ad hoc* scripts for data analysis.
- perl : *ad hoc* scripts and utilites for batch analysis and MGF preparation.
- C/C++ : *speptide* source code.
- Byonic 2.5.6, PeakStudio 7.0 and pMatch v.1.5.0.1: comparsion with *speptide*.

5.2 Data and code availability

The genomic data was deposited to NCBI either as WGS or as SRA submission. The proteomic data (including FASTA used for Mascot search) is available in PRIDE under accession: PXD001481 (Reviewer account details: Username: reviewer50451@ebi.ac.uk; Password: aR3585SN).

Algorithm was implemented as an *ad hoc* software program *speptide* written in C/C++ available with comprehensive manual and examples at: `https://github.com/dimaischenko/speptide`

6 References

Author details
[1] Research Institute of Physical Chemical Medicine, Malaya Pirogovskaya, 1a, 119435 Moscow, Russian Federation.
[2] Moscow Institute of Physics and Technology, Institutskiy pereulok, 9, 141700, Dolgoprudny, Russian Federation. [3] Scientific Research Institute of Gastroenterology, Shosse Entuziastov, 86, 111123 Moscow, Russian Federation.

References

1. Frank, Ari M., et al. "Spectral archives: extending spectral libraries to analyze both identified and unidentified spectra." Nature methods 8.7 (2011): 587-591.
2. Wan, Katty X., Ilan Vidavsky, and Michael L. Gross. "Comparing similar spectra: from similarity index to spectral contrast angle." Journal of the American Society for Mass Spectrometry 13.1 (2002): 85-88.
3. Liu, Jian, et al. "Methods for peptide identification by spectral comparison." Proteome Science 5.1 (2007): 3.
4. Elias, Joshua E., and Steven P. Gygi. "Target-decoy search strategy for increased confidence in large-scale protein identifications by mass spectrometry." Nature methods 4.3 (2007): 207-214.
5. Lam, Henry, Eric W. Deutsch, and Ruedi Aebersold. "Artificial decoy spectral libraries for false discovery rate estimation in spectral library searching in proteomics." Journal of proteome research 9.1 (2009): 605-610.
6. Gupta, Nitin, et al. "Whole proteome analysis of post-translational modifications: applications of mass-spectrometry for proteogenomic annotation." Genome research 17.9 (2007): 1362-1377.
7. Bern, Marshall, Yong J. Kil, and Christopher Becker. "Byonic: advanced peptide and protein identification software." Current Protocols in Bioinformatics (2012): 13-20.
8. Ye, Ding, et al. "Open MS/MS spectral library search to identify unanticipated post-translational modifications and increase spectral identification rate." Bioinformatics 26.12 (2010): i399-i406.
9. Yuen, Denis. "SPIDER: reconstructive protein homology search with de novo sequencing tags." (2011).
10. Shao, Wenguang, et al. "A peptide identification-free, genome sequence-independent shotgun proteomics workflow for strain-level bacterial differentiation." Scientific reports 5 (2015).
